# Encephalic Leukocytoclastic Vasculitis during Treatment with Sunitinib for Renal Cell Carcinoma: A Case Report

**DOI:** 10.3390/medicines8010005

**Published:** 2021-01-11

**Authors:** Maria Massucci, Veronica Mollica, Alessandro Rizzo, Laura Ventrella, Ilaria Maggio, Lisa Manuzzi, Lidia Gatto, Giovanni Brandi, Francesco Massari

**Affiliations:** 1 Division of Oncology, IRCCS Azienda Ospedaliero-Universitaria di Bologna, Via Albertoni—15, 40138 Bologna, Italy; maria.massucci@studio.unibo.it (M.M.); veronica.mollica7@gmail.com (V.M.); ila.mag.88@gmail.com (I.M.); lisamanuzzi@gmail.com (L.M.); fmassari79@gmail.com (F.M.); 2 Maria Cecilia Hospital, Cotignola (RA), 48033 Ravenna, Italy; gammaknife-mch@gvmnet.it; 3 Medical Oncology Department, Bellaria Hospital, Azienda USL of Bologna, Via Altura n.3, 40139 Bologna, Italy; lidia.gatto83@gmail.com; 4 Oncology Unit, Department of Experimental, Diagnostic and Specialty Medicine, Sant’Orsola-Malpighi Hospital, University of Bologna, 40138 Bologna, Italy; giovanni.brandi@unibo.it

**Keywords:** encephalic vasculitis, sunitinib, renal cell carcinoma (RCC), leukocytoclastic vasculitis, paraneoplastic syndrome

## Abstract

Renal cell carcinoma is a malignant tumor that arises in the kidney parenchyma. For many years, sunitinib has represented the mainstay of medical treatment for metastatic renal cell carcinoma. Herein, we present the case of a 66-year-old woman with metastatic clear cell renal carcinoma undergoing treatment with sunitinib for two years that developed encephalic leukocytoclastic vasculitis, probably due to a paraneoplastic syndrome.

## 1. Introduction

Renal cell carcinoma (RCC) is a malignant tumor arising in the kidney parenchyma [1]; from the histological point of view, clear cell renal cell carcinoma (ccRCC) is the most frequent histological type of renal malignancy, accounting for approximately 75–80% of cases [2]. RCC currently represents the seventh most common type of cancer in men and the tenth in women in Western countries, with about 25–30% of patients presenting metastatic disease at diagnosis [3].

The prognosis and treatment of metastatic RCC depends on prognostic scores based on clinical, laboratory, and pathological parameters [4]. For many years, the mainstay of medical treatment for advanced RCC was represented by interferon alfa (IFN α) and interleukin 2 (IL2) [5]; subsequently, other two categories of drugs proved to be more effective: anti-angiogenic agents and mammalian target of rapamycin (mTOR) inhibitors [6]. In recent years, immune checkpoint inhibitors (ICIs) have been used, at first as second-line treatment, and subsequently as front-line treatment options in metastatic RCC (mRCC), where immune-based combinations have profoundly modified the therapeutic algorithm of this malignancy [7].

Sunitinib is an oral, multi-target tyrosine kinase inhibitor (TKI) which has an anti-angiogenic effect through the inhibition of vascular endothelial growth factor (VEGF) and platelet-derived growth factor (PDGF) receptors; for many years, sunitinib has been one of the standard first-line treatments for mRCC [8]. As in the case of other multi-kinase inhibitors, sunitinib has been associated with a wide range of grade 1 to 4 adverse events—including skin disorders, hand–foot syndrome, periorbital edema, hair depigmentation, skin discoloration, subungual splinter hemorrhages, and genital rash [9]. Other manifestations, such as vasculitis, have been rarely reported in patients receiving sunitinib [10,11].

Paraneoplastic syndromes, that are correlated to the production of specific molecules by tumor cells or by the immune response to the tumor, have been described in about 10–40% of patients with mRCC [12]. Herein, we present a case of encephalic vasculitis in a patient undergoing first-line treatment with sunitinib for metastatic ccRCC.

## 2. Cases and Methods

In January 2015, a 66-year-old woman underwent left nephrectomy for localized ccRCC without distant metastases. A computed tomography (CT) scan performed during follow-up in September 2017 showed adrenal metastases; thus, sunitinib 50 mg daily on a 4 weeks on/2 weeks off schedule was started as first-line therapy. With this treatment, the patient achieved a partial response without experiencing important toxicities.

**Figure 1:**
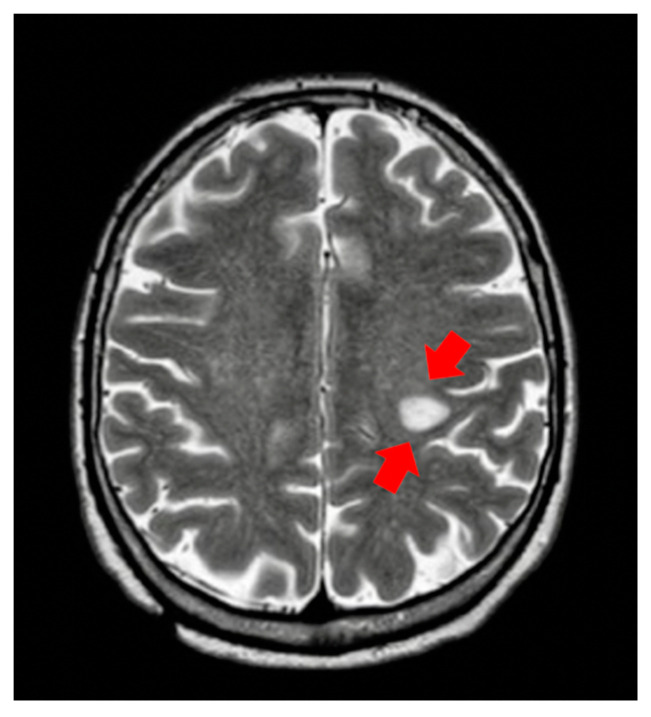
First brain magnetic resonance imaging showing encephalic vasculitis during sunitinib treatment.

A total body CT scan performed two years after the start of treatment showed the appearance of a hypodense area of two centimeters in the left rolandic area. Given the unclear nature of this lesion, brain magnetic resonance imaging (MRI) was performed, with the radiological images showing the probable vasculitic nature of the lesion (Figure 1). The patient was asymptomatic and, in particular, did not present any neurological symptoms. To exclude that vasculitis could be correlated to a paraneoplastic syndrome, we evaluated anti-neutrophil cytoplasmic antibodies (ANCAs) in the blood, which were negative—a finding which supported the hypothesis that the vasculitis could be due to a paraneoplastic syndrome.

**Figure 2:**
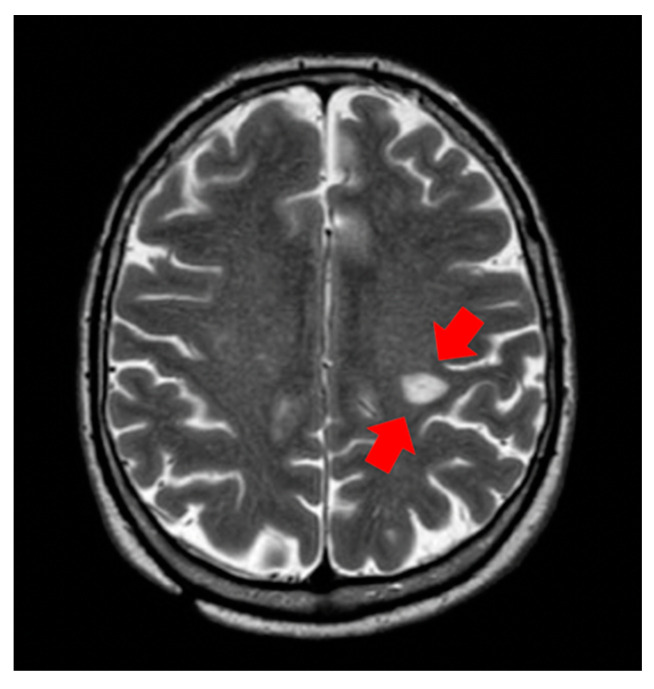
Second brain magnetic resonance imaging performed after discontinuation of sunitinib and steroid therapy.

The patient discontinued sunitinib treatment and was started on steroid therapy. A subsequent brain MRI demonstrated a small reduction of the known brain lesion, probably due to the use of steroids (Figure 2). A total body CT scan showed the stability of previous findings in the other metastatic sites; thus, the patient continued the suspension of sunitinib with a programmed tumor imaging assessment after two months.

## 3. Discussion

Leukocytoclastic vasculitis (LCV) represents a small vessel vasculitis with inflammatory infiltrate composed of neutrophils. The development of LCV is associated with both immune complex deposition and hypersensitivity to a suspected drug. LCV represents a rare complication during anticancer treatments and it can be associated with chronic infection, drugs, and paraneoplastic syndrome. When LCV is diagnosed during cancer treatment, it is very important to determine if the vasculitis is associated with the ongoing therapy to evaluate whether to continue such treatment safely [12].

One hypothesis is that the abnormal production of antibodies and tumor neoantigens lead to the formation of immune complexes that are deposited inside the walls of blood vessels, an event constituting a paraneoplastic syndrome.

Another explanation could be the relationship between a specific drug and vasculitis. Although the mechanism of drug-related LCV development remains unclear, a possible reason is that activated neutrophils transform the drug to an immunogenic product, which activates immune cells to produce ANCAs. This mechanism explains why multispecific ANCAs are common in drug-induced LCV. Notably, in some cases, vasculitis may also occur after a drug dosage increase and after the reintroduction of the suspected drug [13].

Very few cases of TKI-associated vasculitis have been described in the literature [14,15,16]. Some cases of LCV related to the use of epidermal growth factor receptor (EGFR) TKIs and one case related to the use of anaplastic lymphoma kinase (ALK) TKIs have been reported in the literature [17].

Only one case of vasculitis has been described following sunitinib treatment. In this case, the patient developed LCV after three years of sunitinib therapy; the patient was treated with prednisone, achieving complete resolution of the symptoms [18].

## 4. Conclusions

In this report, we present a case of encephalic leukocytoclastic vasculitis, presumably correlated to a paraneoplastic syndrome. Leukocytoclastic vasculitis generally presents as a purpuric rash involving various cutaneous sites; in our case, considering the absence of skin lesions and the location of the metastatic site, the biopsy was postponed, and the diagnosis was based on clinical signs. Notably, it is important to consider the possible correlation between sunitinib treatment and the onset of drug-related LCV that could lead to the interruption of an active treatment for the oncologic disease. Lastly, another important aspect to consider is to differentiate between drug-related LCV and disease progression or paraneoplastic syndromes, in order to guide the decision-making process.
